# Evolutionarily Distant Streptophyta Respond Differently to Genotoxic Stress

**DOI:** 10.3390/genes8110331

**Published:** 2017-11-17

**Authors:** Radka Vágnerová, Alena Lukešová, Martin Lukeš, Petra Rožnovská, Marcela Holá, Jana Fulnečková, Jiří Fajkus, Karel J. Angelis

**Affiliations:** 1 Institute of Experimental Botany, Czech Academy of Sciences, v.v.i., Na Karlovce 1, 16000 Prague 6, Czech Republic; radka.vagnerova@centrum.cz (R.V.); roznovska@gmail.com (P.R.); marcelahola@seznam.cz (M.H.); 2 Biology Centre ASCR, v.v.i., Institute of Soil Biology, Na Sádkach 7, 37005 České Budějovice, Czech Republic; luksa@upb.cas.cz; 3 Institute of Microbiology ASCR, Centrum Algatech, Laboratory of photosynthesis, Opatovický mlýn, 379 81 Třeboň, Czech Republic; lukesm@alga.cz; 4 Institute of Biophysics, Czech Academy of Sciences, v.v.i., Královopolská 135, 61265 Brno, Czech Republic; fulneckova@ibp.cz; 5 Laboratory of Functional Genomics and Proteomics, NCBR, Faculty of Science, Masaryk University, Kotlářská 2, 61137 Brno, Czech Republic; fajkus@sci.muni.cz; 6 Mendel Centre for Plant Genomics and Proteomics, CEITEC—Central European Institute of Technology, Masaryk University, Kamenice 5, 62500 Brno, Czech Republic

**Keywords:** *Physcomitrella patens*, *Klebsormidium*, *Zygnema*, DNA damage and repair, bleomycin, methyl methanesulfonate, ultraviolet light

## Abstract

Research in algae usually focuses on the description and characterization of morpho—and phenotype as a result of adaptation to a particular habitat and its conditions. To better understand the evolution of lineages we characterized responses of filamentous streptophyte green algae of the genera *Klebsormidium* and *Zygnema*, and of a land plant—the moss *Physcomitrella patens*—to genotoxic stress that might be relevant to their environment. We studied the induction and repair of DNA double strand breaks (DSBs) elicited by the radiomimetic drug bleomycin, DNA single strand breaks (SSB) as consequence of base modification by the alkylation agent methyl methanesulfonate (MMS) and of ultra violet (UV)-induced photo-dimers, because the mode of action of these three genotoxic agents is well understood. We show that the *Klebsormidium* and *Physcomitrella* are similarly sensitive to introduced DNA lesions and have similar rates of DSBs repair. In contrast, less DNA damage and higher repair rate of DSBs was detected in *Zygnema*, suggesting different mechanisms of maintaining genome integrity in response to genotoxic stress. Nevertheless, contrary to fewer detected lesions is *Zygnema* more sensitive to genotoxic treatment than *Klebsormidium* and *Physcomitrella*

## 1. Introduction

Responses of living organisms to DNA damage caused by either intrinsic or environmental factors must act promptly and with a high efficiency to preserve integrity and functionality of genetic information. Although algae play an important ecological role and are considered strong indicators of environmental changes, besides ultra violet light (UV), only few studies have investigated their responses to genotoxic stress and DNA damage. To our knowledge, there is only one report on the response of an Arctic alga *Zygnema* to ionizing radiation (IR) [1]. This is why the purpose of this study is to identify and describe differences in sensitivity to induction and response to genotoxic exposure of algae species from two genera—*Klebsormidium* and *Zygnema -* as well as moss *Physcomitrella patens* as representatives of Streptophyta lineages. 

The Streptophyta studied have filamentous morphology, are widespread all over the world and belong to ecologically important organisms, often forming mats or crusts. Algae of both genera live in environments where they have to cope with severe conditions, such as *Zygnema* species occupying hydro-terrestrial habitats [2,3,4] and *Klebsormidium* species inhabiting aero-terrestrial habitats [5,6,7]. For comparison of genotoxic sensitivity, we used in parallel to algae moss, a bryophyte, *P. patens* that is recognized as an elegant model of not flowering plants [8]. Bryophytes represent the oldest living branch in land plant evolution [9] and moss protonemata morphologically resemble algal filaments. 

Species that successfully transitioned from aqueous to terrestrial environments acquired a phenotype adapted to the new conditions. To characterize differences in sensitivity phenotype among *Klebsormidium, Zygnema* and *Physcomitrella*, we took advantage of their morphology and studied their sensitivity to genotoxic treatment in cultures of short filament fragments. The use of cultures with enriched fraction of dividing cells was previously established in the moss *P. patens* [10,11,12]. The clonal propagation of these cells amplifies the potential effects and consequences of incurred DNA damage to plant growth and phenotype. 

To identify differences among studied Streptophyta, we studied their sensitivity and response to three types of genotoxic attacks, predicted as relevant to the conditions of their environment. Firstly, oxidative stress exerted by bleomycin, which mimics the impact of IR by generating a burst of reactive oxygen species (ROS), which interact with DNA and are responsible for induction of solitary as well as clustered DNA lesions [13]. Burst of ROS in plants also accompanies other biotic and abiotic stresses including desiccation or salinity. Secondly, we studied effects of methyl methanesulfonate (MMS), a S_2_N alkylation agent, which attacks DNA bases as an alkylation modifier like nitrosoureas, and similarly naturally occurring nitrosocompounds. Thirdly, as an example of genotoxic physical stress, we used UVC to induce cyclobutene pyrimidine (CPD) and 6′-4′ pyrimidine-pyromidone (6-4PP) photo-dimers. UVC was used instead of UVB to simulate terrestrial solar UV exposure; because the yield and ratio of UVC and UVB induced photo-dimers is identical and the use of UVC is experimentally simpler. In addition, both UVC and UVB radiation are reported to interfere with photosynthesis and thus to attenuate the growth of algal cells [14].

## 2. Materials and Methods

### 2.1. Plant Material

Algae—Six strains from orders Klebsormidiales and Zygnematales were studied. Five axenic strains K101—*Klebsormidium flaccidum* SAG 7.91, K 292—*Klebsormidium elegans* SAG 7.96, K293—*K. flaccidum* SAG 2307, Z181—*Zygnema circumcarinatum* SAG 698-1aZ294—*Zygnema* sp. SAG 698-4 were obtained from Sammlung von Algenkulturen (SAG), University of Goettingen, Germany. Strain Z436—*Zygnema* sp., freshly isolated from a shallow seepage pool in Petunia Bay, Svalbard (High Arctic) was kindly provided by Martina Pichrtova, FS, Charles University, Prague, Czech Republic.

Algae were grown on bold-basal medium (BBM) agar plates [15] at 20–24 μmol photons m^−2^s^−1^ at 20 °C and 14/10 h light/dark regime.

Moss—*P. patens* “Gransden 2004” wild type used in this study was kindly provided by Andrew C. Cuming, CPS, Univ. Leeds, UK. *Physcomitrella* was vegetatively propagated by weekly subculture of homogenized protonemata on routine basal BCD agar medium supplemented with 1 mM CaCl_2_ and 5 mM ammonium tartrate as a biomass on Petri plates overlaid with cellophane in growth chambers with 18/6 h day/night cycle at 22/18 °C [16].

### 2.2. Algal Growth and Phenotype Assays

Biomass of *Klebsormidium* K101 and *Zygnema* Z436 in exponential phase of growth were collected from Petri plates and gently sheared in fresh liquid BBM medium. Aliquots of suspension culture were then treated with indicated concentrations of bleomycin (0–300 μg/mL) and MMS (0–200 mM) in BBM medium for 30 min. After the treatment cultures were washed by 3 cycles of centrifugation and suspension in fresh medium and finally resuspended as a 5-times concentrate of the original volume. 15 μL of untreated or treated suspension were spotted as inocula in sectors on agar plates and grown under continuous light at 25 °C for 14 days. Five spots per concentration sector on plate and 6 replica plates were set for every experiment. Growth of spot inocula was monitored and photo-documented. For microscopy, the algae were cultivated as liquid culture in 24-well microtiter plates, 200 μL of algal suspension of each treatment was transferred into a well and volume was adjusted to 1.5 mL. There were 4 replicas of each treatment. 30 filaments per treatment were reviewed and photographed using Olympus BX 51 microscope equipped with Olympus DP 50 digital camera (Olympus Czech Group sro., Prague, Czech Republic) on the 1st, 7th and 14th day after the treatment. In total, 3 independent experiments were performed for both growth and cell damage monitoring.

### 2.3. Genotoxin Treatments for DNA Damage Quantification

Biomass of vegetatively propagated *Klebsormidium, Zygnema* and *P. patens* were collected from plates, suspended in 8 mL of BBM or BCD media per plate respectively and sheared with a T25 homogenizer (IKA, Staufen, Germany) at 10,000 rpm for two 1-min cycles. Sheared filament culture was left for overnight recovery in a cultivation chamber with gentle shaking at 100 rpm. This preparation yielded suspension cultures of 2–7 cell filaments, which can be distributed by pipetting and collected by filtering through 50 μm mesh Partec CellTrics filters (Sysmex Deutschland GmbH, Norderstedt bei Hamburg, Germany). Adopted from [11].

Bleomycin as Bleomedac inj. (Medac, Hamburg, Germany) and MMS cat. M4016 (Sigma-Aldrich sro., Prague, Czech Republic) were used for genotoxin treatments as freshly prepared solutions from weighed or pipetted reagent in BBM or BCD media, respectively. UVC treatment was carried out in a Hoeffer UVC 500 cross-linker at 254 nm. Irradiation and the following steps until harvest and freezing of samples were performed in the dark or under red illumination to block photolyases, which are activated by blue light (435 nm). 

### 2.4. Detection of DNA Lesions

DNA breaks were measured in DNA of mechanically isolated nuclei by a single cell gel electrophoresis (comet) assay [17]. Nuclear suspension from approximately 100 mg of frozen plant samples was dispersed in melted 0.7% low melting agarose cat. 15510-027 (Life Technologies sro., Prague, Czech Republic) at 40 °C and four aliquots were immediately pipetted in duplicate on two agarose-coated microscope slides, covered with a cover slips and chilled on ice for 1 min to let agarose solidify. After removal of cover slips, slides were immersed in lysis solution (2.5 M NaCl, 10 mM Tris-HCl, 0.1 M EDTA, and 1% sodium N-lauroylsarcosinate, pH 7.6) at room temperature for 1 h to dissolve cellular membranes and remove attached proteins. The whole procedure from chopping plant biomass by razor blade to placement into lysis solution took approximately 3 min.

After lysis, slides were either first incubated for 20 min in unwinding solution (0.3 M NaOH, 5 mM EDTA, pH 13.5) to partially unwind the DNA double helix and reveal single strand breaks (SSBs) (A/N protocol) [17,18], or, for detection of double strand breaks (DSBs), were directly processed without unwinding (N/N protocol), twice equilibrated for 5 min in tris-acetate (TA) electrophoresis buffer (100 mM Tris, 300 mM sodium acetate, pH 9) to remove salts and detergents [19,20]. After 3 min of electrophoresis at 1 V/cm, slides were washed in 70% and 100% ethanol and air dried. UV dimers were detected as SSBs induced at the sites of dimers by digestion with CPD specific T4 endonuclease V (T4EndoV) prior to alkaline unwinding in the A/N protocol. T4EndoV was prepared as a crude lysate of an overexpressing bacterial strain [21] and applied as described in Holá et al., 2015 [11].

Comets were stained with SYBR Gold (Molecular Probes/Invitrogen), viewed in epifluorescence with a Nikon Eclipse 800 microscope (Nikon CEE GmbH, Prague, Czech Republic) and blind-evaluated by the LUCIA Comet Assay (LIM Inc., Prague, Czech Republic). The fraction of DNA in comet tails (% T DNA) was used as a measure of DNA damage. The percentage of remaining damage (% DSB remaining) after a given repair time (t_x_) is defined as:

$$\%\;\mathrm{damage}\;\mathrm{remaining}\;\left(\mathrm{tx}\right)=\frac{\mathrm{mean}\;\%\;T\;\mathrm{DNA}\left(tx\right)-\mathrm{mean}\;\%\;T\;\mathrm{DNA}\;\left(\mathrm{control}\right)}{\mathrm{mean}\;\%\;T\;\mathrm{DNA}\left(t0\right)-\mathrm{mean}\;\%\;T\;\mathrm{DNA}\;\left(\mathrm{control}\right)}\times 100$$

Data in this study were obtained from at least three independent experiments, in which 25 comets were evaluated in each of four independent gels providing at least 300 comets analyzed per experimental point. Repair kinetics was analyzed by Prism v.5 program (GrafPad Software Inc., La Jolla, CA, USA).

## 3. Results

### 3.1. Preparation of Streptophyta for Genotoxicity Testing

**Figure 1:**
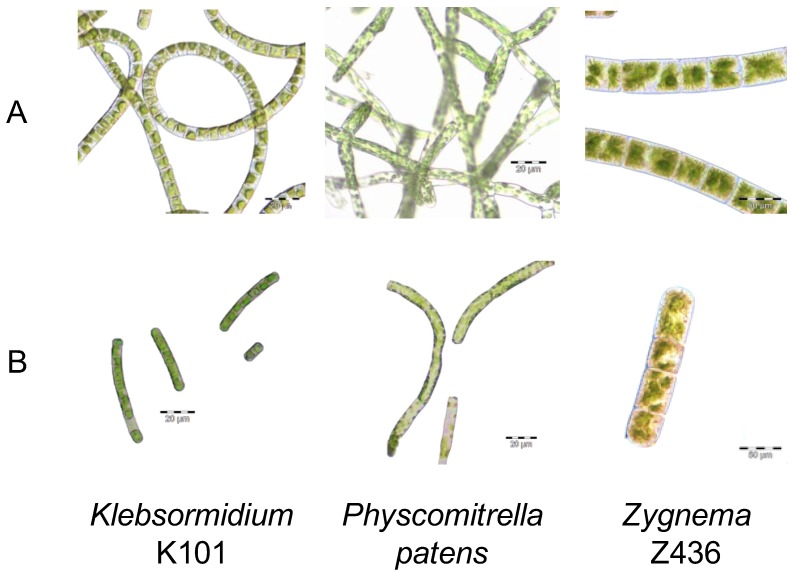
Fragmentation of the algae *Klebsormidium flaccidum* K10, *Zygnema* sp. Z436 and of the moss *Physcomitrella patens*. Regularly growing algae and moss on plates (**A**) were sheared to smaller fragments of app. 2–7 cells in length (**B**).

To obtain comparable conditions for algal strains *Klebsormidium* and *Zygnema* and moss *P. patens*, we carried out all experiments in liquid culture, which was left to recover for 1 day after mechanical shearing. According to previous experience with *Physcomitrella* [12], the use of short fragments avoids differences due to growth stage and history of culture. In regenerating moss filaments of 2–7 cells length, are up to 50% of the cells potentially mitotically active apical cells, what is in number close to number of cells in algal fragments where all cells have potency to divide. Outcome of shearing is illustrated on Figure 1 and is clearly similarly effective in all strains.

### 3.2. Sensitivity of Klebsormidium and Zygnema to Genotoxic Treatment

**Figure 2:**
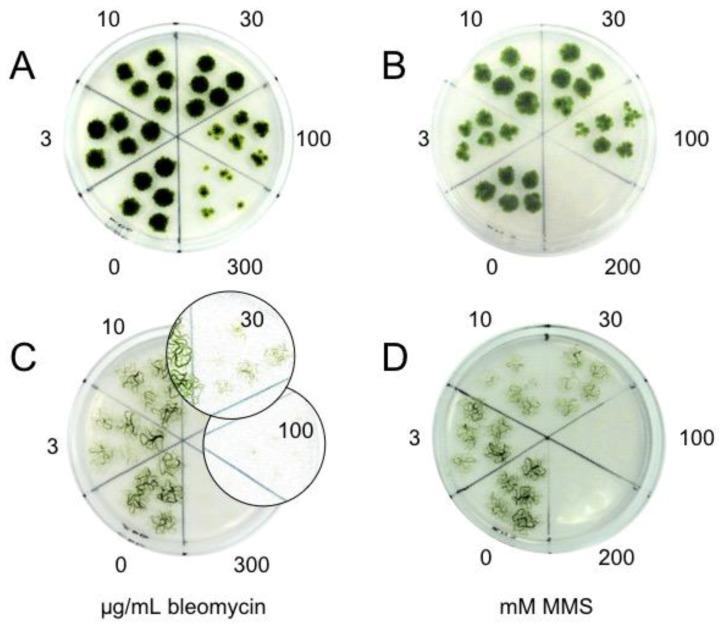
Sensitivity of the *K. flaccidum* K101 (**A,B**) and *Zygnema* sp. Z436 (**C,D**) to bleomycin (**A,C**) and methyl methanesulfonate (MMS) (**B,D**) treatment. The cultures of fragmented algae were treated for 30 min in liquid bold-basal medium (BBM) with the indicated concentrations of bleomycin and MMS, then spot inoculated on BBM agar plates and grown under continuous light for 2 weeks. Few filaments of *Zygnema* sp. Z436 survived or developed from surviving cells treated with 30 and 100 μg bleomycin/mL. To visualize surviving individual filaments the enlarged sections with increased contrast are inserted in panel C. No viable cells were found at 300 μg bleomycin/mL, as well as at 100 and 200 mM MMS. Less sensitive *Klebsormidium* K101did not survive only the highest, 200 mM MMS treatment.

The recovery of the algae *K. flaccidum* K101 and *Zygnema* sp. Z436 treated for 30 min with different concentrations of bleomycin and MMS was monitored as growth of spot inocula over a 2-week period (Figure 2). Algal growth was unimpaired by bleomycin treatment at concentrations of 30 μg/mL in *K. flaccidum* K101 or 10 μg/mL in *Zygnema* sp. Z436. At higher concentrations (100–300 μg bleomycin/mL), the growth of *K. flaccidum* K101 was inhibited, whilst for *Zygnema* sp. Z436 the concentration 30 μg bleomycin/mL was already severely detrimental and at 100 μg/mL was almost lethal. This indicates nearly one order of magnitude higher sensitivity of *Zygnema* sp. Z436 to destruction by bleomycin. Nevertheless, few surviving filaments (enlarged sections in Figure 2C) are still present in the foci of otherwise ‘dead’ cells. Similarly, a higher sensitivity of *Zygnema* sp. Z436 to MMS than *K. flaccidum* K101 is displayed in Figure 2B,D.

**Figure 3:**
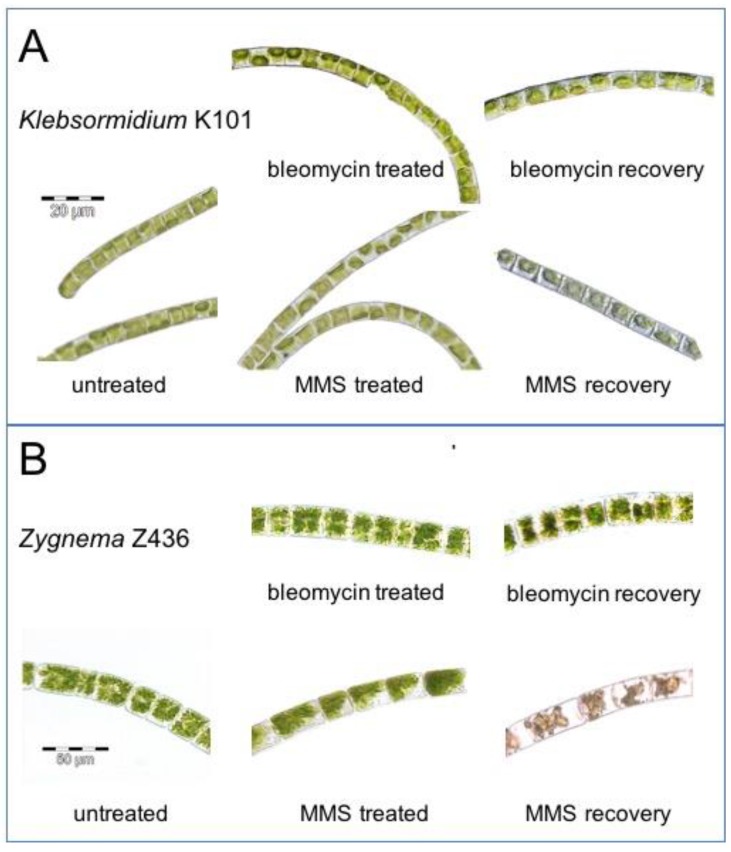
Effect of genotoxic treatment on morphology of algal cells. The suspension cultures of *K. flaccidum* K101 (**A**) and *Zygnema* sp. Z436 (**B**) in bold basal medium (BBM) were treated 30 min with 100 μg bleomycin/mL or 100 mM MMS, followed by 7 days recovery cultivation in BBM medium under continuous light. No morphological changes are visible in *Klebsormidium* K101 cells first day after bleomycin or MMS treatment depicted as treated cells. Certain tendency to chlorophyll bleaching could be seen in MMS treated samples of *Klebsormidium* K101 cells after 7 days of recovery. Irreversible damage of *Zygnema* sp. Z436 cells due to disintegration of organelles and chlorophyll degradation is manifested after 7 days recovery from MMS treatment.

The morphology of cells of *K. flaccidum* K101 (Figure 3A) and *Zygnema* sp. Z436 (Figure 3B) also differed in response to bleomycin and MMS treatment. No apparent damage of algal cells was observed one day after genotoxin treatment, including the highest concentrations, except visible shrinkage of chloroplasts in some *Zygnema* cells treated with 100 mM MMS. During following 7 days of cultivation no recovery from cellular damage was observed and all cells in filaments exhibited irreversible morphological changes such as disintegration of organelles, especially of chloroplast and chlorophyll degradation. Less cell damage was observed in *Zygnema* sp. Z436 after bleomycin treatment, often with presence of damaged and not damaged cells in one filament. By contrast to *Zygnema* fewer morphological changes were seen in *K.* *flaccidum* K 101 cells following bleomycin and MMS treatments.

The sensitivity of *P. patens* to bleomycin treatment and UV irradiation has been previously described [12].

### 3.3. DNA Damage in Klebsormidium, Zygnema and Physcomitrella

**Figure 4:**
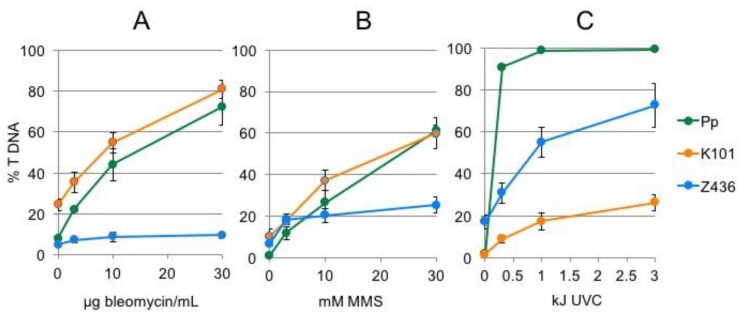
The dose-responses of algae and moss to genotoxic treatment. The suspension cultures of *K. flaccidum* K101, *Zygnema* sp. Z436 and *Physcomitrella* (Pp)were treated with 0–30 μg/mL bleomycin (**A**) and 0–30 mM MMS for 1 h (**B**). The same cultures spotted on agar plates were irradiated with 0–3 kJ UVC (**C**). Upon treatment, the culture was blotted dry and immediately frozen in liquid nitrogen prior extraction of nuclei and analysis of DNA damage by the comet assay. The extent of DNA damage is indicated by the proportion of fragmented DNA in the ‘comet tails’ (% T DNA). Double strand breaks (DSBs) were followed as an end point of bleomycin treatment, single strand breaks (SSBs) as an endpoint of MMS treatment and DNA pyrimidine (CPDs) as an endpoint of UVC irradiation detected as T4EndoV sensitive sites.

To characterize differences in vulnerability to DNA damage between algae and moss, we used comet assay and treatments with bleomycin to study induction and repair of DSBs, MMS to study induction and repair of SSBs and UVC irradiation to study induction and dark repair of CPDs (Figure 4). Bleomycin and MMS treatment induces several times more DNA lesions in *Klebsormidium* K101 and *Physcomitrella* than in *Zygnema* Z436 (Figure 4A,B).

**Figure 5:**
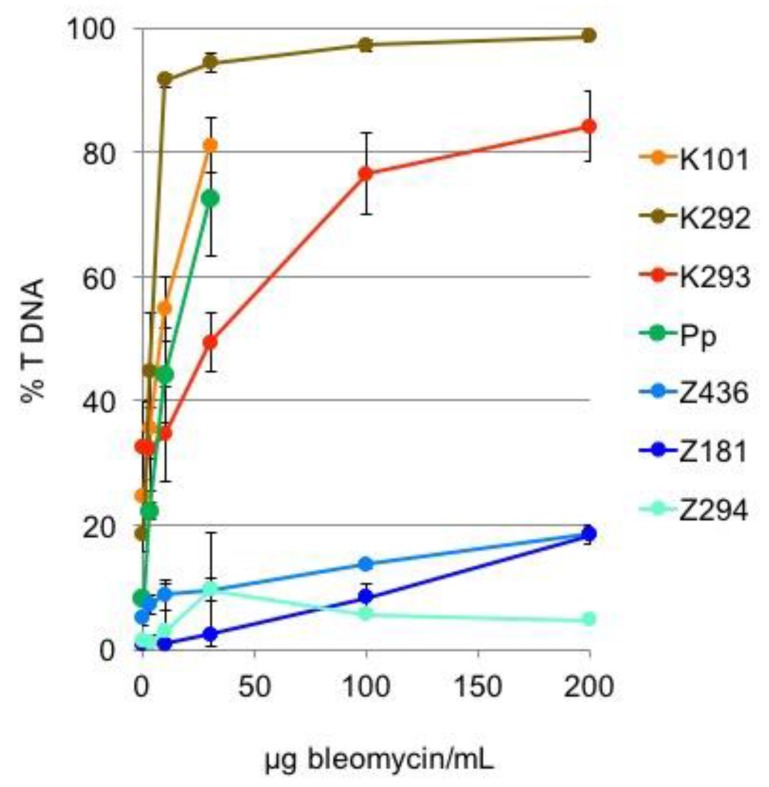
Bleomycin dose-response of *Klebsormidium* and *Zygnema* species. The cultures of *K. flaccidum* K101 (heavy orange), *K. elegans* K292 (brown), *K. flaccidum* K293 (red), *Zygnema* sp. Z436 (mid blue), *Z*. *circumcarinatum* Z181 (dark blue), *Zygnema* sp. Z294 (cyan), and *Physcomitrella* (Pp, green) were treated with an extended concentration range of bleomycin (0–200 μg/mL) for 1 h and analyzed for induction of DSBs.

To rule out distinct responses to genotoxic treatment unique to the *K. flaccidum* K101 and *Zygnema* sp. Z436 strains, we compared the sensitivity of three strains of each genera to induction of DSBs. We tested two strains of *K. flaccidum* denoted K101 and K293, and *K. elegans* K292. Zygnematales were represented by two strains of *Zygnema sp*. Z436 and Z294, and by *Z. circumcarinatum* Z181. The sensitivity of *Physcomitrella* to bleomycin induction of DSBs is similar to that of *Klebsormidium* strains (Figure 5).

*Physcomitrella* and *Klebsormidium* share a similar vulnerability and response of cells to genotoxin treatment in contrast to distinct *Zygnema*. Contrary to bleomycin and MMS treatment *Zygnema* sp. Z436, and particularly *Physcomitrella*, are far more sensitive to the induction of CPDs than *K. flaccidum* K101 (Figure 4C).

### 3.4. Double Strand Break Repair

**Figure 6:**
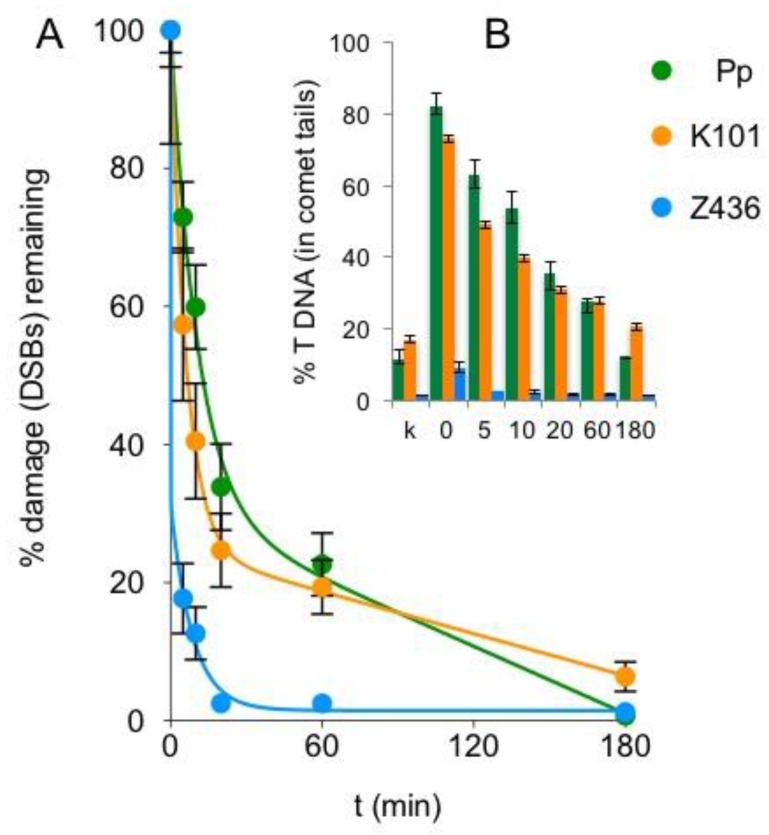
Kinetics of DSBs repair. The cultures of *Klebsormidium flaccidum* K101 (orange), *Zygnema sp* Z436 (blue) and *Physcomitrella* (green) were treated for 1 h with 30 μg bleomycin/mL and allowed to recover for the indicated times. The fraction of DNA measured in comet tails % T DNA (embedded graph B) was normalized to 100% damage after treatment (t = 0), to highlight differences among each strain. The graph plot A was generated as approximation of two-phase decay kinetics through the experimental points by the Prism v.5 program.

Repair kinetics was studied in 1-day regenerating cultures (Figure 6). The repair of DSBs induced by 30 μg bleomycin/mL could be approximated by two-phase decay kinetics in *Klebsormidium* K101, *Zygnema* sp. Z436 and *Physcomitrella* strains. The observed half-life of DSBs during the first, rapid phase of the repair is 4.1 min. in *Physcomitrella* and 5.8 min in *K. flaccidum* K101, whilst only 1.9 min in *Zygnema* sp. Z436. This indicates that while *K. flaccidum* K101 and *Physcomitrella* repair DSBs at approximately same rate, *Zygnema* sp. Z436 repairs DSBs more than twice as fast. 

## 4. Discussion

### 4.1. Response to Genotoxic Treatment

Transition from water to land and changes from wet to arid environments challenge these species through the accompanying salt and osmotic stress and exposure to environmental inorganic salts, heavy metals and chemicals, some of which are currently widespread as man-made pollutants. In parallel they are also exposed to such physical stresses as extreme temperature fluctuations and exposure to IR and UV radiation. All these factors cause direct damage of DNA and impair cellular functions and this is why plants consequentially evolved multiple mechanisms to avoid, tolerate, or repair DNA damage. In this study, we focused on the immediate responses of plants. To provide uniform conditions for the genotoxic testing of the selected streptophyte species, we used culture of small fragments. The use of small vs. long fragments was devised to study kinetic of DSB repair in apical versus differentiated cells of *Physcomitrella* protonema [12]. In this study, we used the same approach for *Klebsormidium* and *Zygnema* because their algal filament morphology is similar moss protonemata. Of course, there is an obvious difference in polar growth of moss protonema with possible branching of internal cells in contrast to algal filaments that retain the undifferentiated capacity of every cell within filament to divide. In the small fragments the differences in numbers of potentially dividing cells are limited and comparable at the start of experiment. Nevertheless, these conditions represent an experimental approximation and might not necessarily fully reflect natural conditions [22].

As is evident from Figure 1, the cell size of *Physcomitrella* and *Klebsormidium* is closer than that of *Zygnema*. The possible explanation of this structural difference might arise from their natural habitats and evolutionarily developed tolerance to desiccation. The abundance and specific localization of callose correlates with the higher desiccation tolerance of *Klebsormidium* when compared with *Zygnema* [23]. The distribution of callose within the cells provides an explanation for the frequent occurrence of *Klebsormidium* strains or species in hot and cold deserts, which are characterized by low water availability and other stressful conditions [24], whereas *Zygnema* is better adapted to Arctic and Antarctic desiccation. The state of recovery from genotoxic stress after 7 days (Figure 3) shares similarities with desiccation stress [23,25,26,27]. Morphological changes occur over time and do not revert, indicating the induction of permanent changes. This process takes several days and in *Zygnema*, a senescent phenotype rapidly leads to necrosis in all cells. After an acute (30 min) exposure to genotoxic treatment, algae do not form akinete or pre-akinete cells as they do following desiccation stress indicating a terminal fate without possible recovery. Pre-akinetes are crucial for the aero-terrestrial lifestyle of *Zygnema* [25,28]. The hypersensitive cell morphology phenotype is more strongly manifested in *Zygnema* sp. Z436 than in *K. flaccidum* K101 after bleomycin treatment. This observation is also confirmed by growth tests of inocula of treated algal cultures, where *K. flaccidum* K101, but not *Zygnema* sp. Z436 survives on plates despite treatment with 3-fold higher concentrations 300 vs. 100 µg/mL of bleomycin and 100 vs. 30 mM MMS, respectively (Figure 2).

### 4.2. DNA Damage and Repair

In contrast to the higher sensitivity of *Zygnema* Z436 over *Klebsormidium* K101 in growth tests (Figure 2), *Zygnema* Z436 shows far less vulnerability to induction of DSBs by bleomycin and of SSBs by MMS treatment (Figure 4). As depicted in Figure 5, different sensitivities to genotoxic treatment are not solely associated with the *K. flaccidum* K101 and *Zygnema* sp. Z436 strains, but are typical for the whole *Klebsormidium* and *Zygnema* groups. Additionally, repair rates of induced DSBs are 2- to 3-fold faster in *Zygnema* than in *Klebsormidium* and *Physcomitrella*. 

Choi et al. [1] recently described an effect of γ-irradiation of Arctic *Zygnema* with doses up to 5 kGy and found that the photosynthetic efficiency markedly decreased, whereas antioxidant capacity significantly increased. Proteomic analysis using 2D electrophoresis combined with mass spectrometry allowed reliable identification of upregulated proteins by peptide fingerprinting. Identified among the upregulated proteins were DNA ligase and ATP-dependent DNA helicase (KU80 subunit), both related to DSBs repair by Non-Homologous End Joining (NHEJ) pathway, and this is why authors concluded that upregulation of DNA repair-related proteins may be an underlying mechanism of radioresistance against oxidative stress caused by ionizing radiation.

Furthermore, upon desiccation *Zygnema* showed also an induction of stress protection mechanisms such as ROS scavenging and DNA repair. DNA damage, which is linked to ROS formation, was counteracted by a strong upregulation of repair enzymes. For example, plant orthologue of Nijmegen breakage syndrome gene (*NBS1*), which is a part of MRE11-RAD50-NBS1 (MRN) complex involved in recognition and repair of DSBs, was highly induced with 9.7-fold change, suggesting a higher risk of DNA damages associated with desiccation. Moreover, a number of other stress related molecules are produced, that is, chaperones such as late embryogenesis abundant (LEA) proteins, proteins involved in ROS scavenging and DNA repair proteins [27]. Contrary to *Zygnema* in *Klebsormidium* desiccation induced down-regulation of transcripts and complex regulation patterns. Pathways, which were strongly down-regulated, were mainly involved in integrative cellular functions, such as cell division and DNA repair mechanisms [26]. This suggests that at least from a transcriptomic point of view *Zygnema* and *Klebsormidium* have differently controlled stress response.

Direct measurement of damage in genomic DNA and its repair is a good estimate how particular cells are able to convert a non-instructional to an instructional genetic template and thus proceed through the cell cycle. Lesions induced in DNA lead either directly to DNA inactivation as a template or indirectly by increased mutagenesis due to error-prone repair in numerous genes critical for normal development, which are then manifested as a hypersensitive phenotype.

In the first alternative, DNA replication machines of algal or moss cells are challenged by lesions induced within DNA. It can be speculated that drugs targeting DNA induce more DNA lesions in dividing cells because the replicating DNA expose to the drugs in a higher chance than the tightly packed DNA in cells not dividing. The less accumulation of DNA lesions in *Zygnema* cells may due to the immediate death induced by drug, that is, *Zygnema* cells could not repair the strand-breaks, and the replication machine is sensitive to strand-breaks. It stalls the DNA replications and finally leads to cell death. The DNA replication complex in *Klebsormidium* and *Physcomitrella* cells might less sensitive to the drugs so the DNA replication and cell division are not inhibited by the drugs. Since the DNA keeps replicating, strand-breaks accumulate in cells and present an apparent high percentage of damaged DNA.

In the second alternative, DNA lesions are repaired, and the intact template restored, but at the price of alteration of genetic information, leading to accumulation of deleterious mutations incompatible with normal growth and development. Individual cells of an algal filament acquire a different spectrum of DNA lesions and mutations during their error-prone repair, and while the majority of cells ultimately undergo necrosis and die, any surviving cell with no or only tolerated mutation in vital genes could regenerate and perpetuate. This process might account for seldom-occurring surviving cells in foci of *Zygnema* inocula treated with high concentration of bleomycin (insert in Figure 2).

We have previously described this phenomenon in various DNA repair mutants of *Physcomitrella*, where sensitive phenotype was an outcome of increased mutagenesis due to alternative error-prone repair rather than defect of repair itself [10,11,12]. Here it should be noted that in *Zygnema* the upregulated NHEJ pathway by γ-irradiation [1] is a notoriously error-prone repair pathway.

Nevertheless, it is difficult to reconcile observed differences because as we suggested above, it may reflect different routes that evolved to offset stress conditions—drought as well as genotoxic exposure. This is why the different capabilities of *Klebsormidium* and *Physcomitrella* vs. *Zygnema* that evolved due to varied habitat conditions might represent, on a molecular level, the evolution of different induction patterns and utilization of repair pathways in response to genotoxic stress.

### 4.3. Response to UVC

UVC induces CPDs with the same efficiency as terrestrially more relevant UVB, which can be effectively monitored as SSBs by the comet assay following digestion with the CPD specific T4EndoV. When compared to bleomycin and MMS treatment, *Zygnema* Z436, *Klebsormidium* K101 and *Physcomitrella* differed in their order of sensitivity to UVC (Figure 4). The reason might be that all three organisms adapted to different habitats with different need for protection against high UV irradiance and desiccation. Regular exposure of aero-terrestrial *Klebsormidium* species to stress factors need more effective adaptation mechanisms than hydro-terrestrial *Zygnema* species, which are exposed to strong UV occasionally, such as during dry season after desiccation of water bodies.

Unlike *Klebsormidium* species, which have to cope daily with rapid changes of UV exposure in a vegetative state, Arctic and Antarctic *Zygnema* species need to form pre-akinetes, hardened by slow desiccation, to be able tolerate severe ecological stresses including UV irradiation [7]. It is difficult to find an explanation on the DNA level, nevertheless, our results are consistent with recently published results of the high tolerance of various *Klebsormidium* species to UV radiation determined as optimum quantum yield of photosystem II (PSII) as well as the capability to synthesize and accumulate mycosporine—like amino acids (MAAs) as ‘sunscreens’ to enable their aero-terrestrial lifestyle [29]. As we tested only the induction of CPDs, the most likely explanation is different protection by UV-shielding, for example due to the presence of phenolic compounds, as has been reported in *Zygnema* [3]. In contrast, the moss *P. patens* usually occupies and survives in shaded areas and this might be why it did not develop efficient passive photo-protection and thus suffers massive induction of DNA damage. We have recently reported high rate of mutagenesis in *Physcomitrella* due to efficient error-prone bypass of photo-dimers leading to C→T transitions [11]. Whether this mechanism is relevant also in *Zygnema* has to be established.

## 5. Conclusions

We characterized the responses of the green algae *Klebsormidium* and *Zygnema* and of the moss *P. patens* to induction of DSBs by bleomycin, to DNA base alkylation by MMS and to formation of CPDs after UV irradiation. These genotoxic agents with well-characterized modes of action were used to probe the vulnerability of these species to the induction and removal of DNA damage. The *Klebsormidium* species and *Physcomitrella* show a similar sensitivity toward the induction of DNA lesions and of DSB repair. In contrast *Zygnema* species are less sensitive to the induction of DNA damage and exercise a high rate of DSB repair. Nevertheless, contrary to fewer lesions in DNA, *Zygnema* is more sensitive to genotoxic treatment than *Klebsormidium* and *Physcomitrella.*
